# Treatment of Toxic Epidermal Necrolysis With Immunoglobulins in a Burn Center

**DOI:** 10.1186/2197-425X-3-S1-A493

**Published:** 2015-10-01

**Authors:** L Cachafeiro Fuciños, A Agrifloglio, E Herrero de Lucas, MJ Asensio, M Sanchez Sanchez, A García de Lorenzo, IdiPaz

**Affiliations:** Hospital La Paz, Madrid, Spain

## Introduction

Toxic epidermal necrolysis is a serious infrequent skin disease, usually secondary to drug. It is associated with high morbidity and mortality, hence the importance of early detection and appropriate treatment, although currently the most effective treatment remains controversial.

## Objectives

The aim of our study was to evaluate the efficacy of immunoglobulin therapy in patients admitted in a Critical Burn Unit with SJS / TEN.

## Methods

A prospective study has been developed, it included all patients over 16 years old with diagnosis of Stevens-Johnson Syndrome (SJS) and Toxic Epidermal Necrolysis (TEN) that were admitted to the Burns Critical Unit from Hospital Universitario La Paz, since June 2007 until June 2014. a protocol with a multidisciplinary team was established. the diagnosis was confirmed by biopsy and all of them were treated with immunoglobulins 0.75 g / kg / dx 4 days ( ± 200 grams). Date collected were demographic data, the surface of epidermal detachment, causative drug, SCORTEN, length of stay, complications and mortality.

## Results

During this period 24 patients were admitted in our unit, 2 with a diagnosis of SJS, 2 with SJS-TEN overlap, and 20 with TEN. Mean age was 51 ± 13 (16-89). 17 patients (71%) were men. the average stay was 11 ± 5 days (2-90). the average surface of epidermal detachment was 70%, but 13 patients had affected more than 90%. SCORTEN at 24h and 72h was 3. the mortality was 25% (six patients), and the complications that we found were distributive shock, ARDS and renal failure requiring CVVHDF.

## Conclusions

In our study we have seen that the mortality of our patients is lower than expected based on the SCORTEN score (25 % vs 35.3%). Immunoglobulins could be a good option to treat Toxic Epidermical Necrolysis, considering they are critical ill patients, with a large body surface area affected and with a high morbi-mortality. Although a multicenter and randomized study is necessary to establish the best treatment for SSJ/TEN.

**Figure 1:**
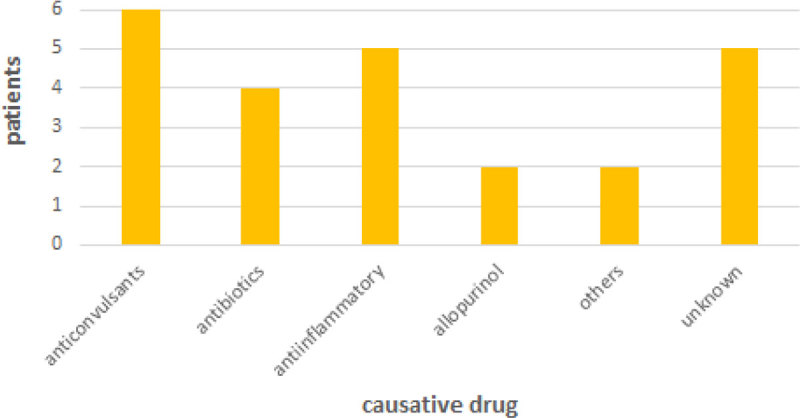
Figure 1
